# Homologous electron transport components fail to increase fatty acid hydroxylation in transgenic *Arabidopsis thaliana*

**DOI:** 10.12688/f1000research.2-203.v2

**Published:** 2013-11-13

**Authors:** Laura L. Wayne, John Browse

**Affiliations:** 1 Institute of Biological Chemistry, Washington State University, Pullman, Washington 99164-6340, USA; 2 Dow AgroSciences, Indianapolis, IN 46268, USA

**Keywords:** Arabidopsis, cytochrome b5, cytochrome b5 reductase, electron transport chain

## Abstract

Ricinoleic acid, a hydroxylated fatty acid (HFA) present in castor ( *Ricinus communis*) seeds, is an important industrial commodity used in products ranging from inks and paints to polymers and fuels. However, due to the deadly toxin ricin and allergens also present in castor, it would be advantageous to produce ricinoleic acid in a different agricultural crop. Unfortunately, repeated efforts at heterologous expression of the castor fatty acid hydroxylase (RcFAH12) in the model plant *Arabidopsis thaliana *have produced only 17-19% HFA in the seed triacylglycerols (TAG), whereas castor seeds accumulate up to 90% ricinoleic acid in the endosperm TAG. RcFAH12 requires an electron supply from NADH:cytochrome b5 reductase (CBR1) and cytochrome b5 (Cb5) to synthesize ricinoleic acid. Previously, our laboratory found a mutation in the *Arabidopsis CBR1* gene, *cbr1-1*, that caused an 85% decrease in HFA levels in the RcFAH12 *Arabidopsis *line. These results raise the possibility that electron supply to the heterologous RcFAH12 may limit the production of HFA. Therefore, we hypothesized that by heterologously expressing RcCb5, the reductant supply to RcFAH12 would be improved and lead to increased HFA accumulation in *Arabidopsis *seeds. Contrary to this proposal, heterologous expression of the top three RcCb5 candidates did not increase HFA accumulation. Furthermore, coexpression of RcCBR1 and RcCb5 in RcFAH12 Arabidopsis also did not increase in HFA levels compared to the parental lines. These results demonstrate that the *Arabidopsis *electron transfer system is supplying sufficient reductant to RcFAH12 and that there must be other bottlenecks limiting the accumulation of HFA.

## Introduction

Ricinoleic acid ((9Z,12R)-12-hydroxyoctadec-9-enoic acid), a hydroxylated fatty acid (HFA), is an important industrial feedstock for products such as lubricants, polyamide 11 (Nylon 11), coatings, inks, surfactants, emulsifiers, and biodiesel ^1^. Castor oil is composed of up to 90% ricinoleic acid that is stored in the developing endosperm of the castor plant ( *Ricinus communis*) in the form of triacylglycerol (TAG). However, castor seeds also contain the deadly toxin ricin, as well as a 2S albumin that is a major allergen ^2, 3^. These proteins, as well as the poor agronomic characteristics of the plant, make castor unsuitable as a major crop. While United States farmers are reluctant to grow castor, the demand for castor oil is increasing. Currently, industrial demand for castor oil is met from plants grown and manually harvested in India, China, and Brazil. Therefore, it would be advantageous to produce ricinoleic acid in a suitable crop species, such as canola, soybean, or camelina that lack ricin and the 2S albumin and can be widely grown in the United States. Furthermore, the knowledge gained from studying the biosynthesis of HFA in transgenic plants could be used to explore the synthesis of other novel fatty acids, such as epoxy fatty acid or conjugated fatty acids, which are made through similar biosynthetic mechanisms that rely on catalysis by modified desaturase enzymes ^1^.

The castor fatty acid hydroxylase (RcFAH12) is a diiron, mixed-function oxidase responsible for the synthesis of ricinoleic acid from oleate (18:1) on phosphatidylcholine (PC) in the endoplasmic reticulum membrane ^4, 5^. The reaction mechanism requires transfer of two electrons from NADH through cytochrome b5 reductase (CBR1) and cytochrome b5 (Cb5) and then to the catalytic enzyme RcFAH12, which oxidizes 18:1 to 18:1-OH with the reduction of oxygen to water ^6, 7^.

Repeated attempts to express a *RcFAH12* cDNA in *Arabidopsis* under the control of seed-specific promoters have yielded a maximum of only 17% HFA in seed oil ^8, 9^. Ricinoleic acid has also been produced in tobacco but at a very low yield ^7^. In *Arabidopsis*, ricinoleic acid can be further desaturated to densipolic acid (18:2-OH) or elongated to lesquerolic acid (20:1-OH) and auricolic acid (20:2-OH) by the fatty acid elongase FAE1 and associated enzymes ^8^. To reduce the range of HFAs produced, we transformed *RcFAH12* into the *fae1* background ^10^ under the control of the seed-specific phaseolin promoter ^11, 12^. One of the lines generated, CL37, has a total HFA accumulation (18:1-OH plus 18:2-OH) of 17–19% ^13^ and has been chosen for experimental investigations aimed at increasing the accumulation of HFA in the seed oil.

In CL37 plants, we assume that RcFAH12 relies on the endogenous *Arabidopsis* electron transfer components, but these may not interact as efficiently with the RcFAH12 protein as do the components of the castor electron transfer system. The *Arabidopsis* fatty acid desaturases FAD2 and FAD3, which convert 18:1 into linoleic acid (18:2) and 18:2 into α-linolenic acid (18:3), respectively, are also diiron proteins that require reductant from CBR1 and Cb5 ^14– 17^. Previously, we have shown that a hypomorphic mutation in the *CBR1* gene ( *cbr1-1*) led to an 85% decrease in HFA levels in RcFAH12 *Arabidopsis* seeds, but much smaller decreases in 18:3 and 18:2 ^15^. We concluded that, in the *cbr1-1* mutant, the very substantial decrease in HFA was caused by an inadequate supply of electrons reaching the hydroxylase via Cb5, demonstrating that the *Arabidopsis* cytochrome b5 electron supply was critical to the activity of RcFAH12. It is possible that the decreased accumulation of HFA is a result of poor interaction between heterologous RcFAH12 enzyme and the endogenous *Arabidopsis* Cb5 proteins. Furthermore, in a separate study, we have shown that the activity of a *Tetrahymena thermophila* desaturase expressed in yeast is limited by weak interaction with the endogenous yeast Cb5. Activity of this desaturase was increased nearly tenfold by coexpression with a *T. thermophila* Cb5 protein ^18^. Together, these results suggest that productive protein-protein interactions within the endoplasmic-reticulum electron transport chain are critical to supporting hydroxylase and desaturase activities.

To test the possibility that electron supply to the RcFAH12 enzyme may be a constraint on hydroxylase activity and accumulation of HFA in seeds of the CL37 line, we set out to identify castor genes encoding components of the endoplasmic reticulum electron transport chain and express them in the CL37 line, under control of seed-specific promoters. We tested three *RcCb5* genes, and we also expressed each of these alongside the gene encoding castor cytochrome b5 reductase ( *RcCBR1*). None of the gene combinations that we tested in a total of 270 independent transgenic lines provided any substantial increase in seed HFA content. Our results strongly indicate that electron supply is not limiting the activity of the RcFAH12 hydroxylase in these transgenic *Arabidopsis* lines.

## Methods

### Plant materials and growth conditions

Seeds from *Arabidopsis* (ecotype Columbia-0) containing the *RcFAH12* transgene line CL37 ^13^ were stratified for 2–3 d at 4°C and germinated on 1× MS medium (Sigma-Aldrich) supplemented with 1% (w/v) sucrose and 0.75% agar. Seeds were germinated with a 16 h day/8 h night cycle at 22°C with 80 µmol m ^-2^ s ^-1^ light. Ten to 12 day old seedlings were then transferred to soil and grown under 24 h light at 22°C with 200 µmol m ^-2^ s ^-1^ light at rosette height in a growth chamber or under a 16 h day/8 h night cycle at 22°C with greater than 300 µmol m ^-2^ s ^-1^ light in the greenhouse.

### Real-time PCR

RNA was previously extracted by Dr. Chaofu Lu of Montana State University from castor developing endosperm, when the RcFAH12 is most highly expressed ^19^. This RNA was then used to synthesize cDNA using Superscript III (Life Technologies) reverse transcriptase. Quantitative reverse transcription PCR (RT-PCR) was then performed with a MX3005P QPCR System (Stratagene) using the DNA binding fluorescent dye SYBR Green I (Life Technologies) and five sets of primers, amplifying full-length cDNA from each gene, see Supplementary Table 1 for list of primers. The collected data was then normalized to the *RcACTIN* control gene and the relative fold change was calculated (=1/(2^(experimental-control))).

### Identification of castor genes and cloning procedures

Castor genes were subcloned from a castor seed cDNA library ^19^ or cloned using castor endosperm RNA extracted by Dr. Lu. Reverse transcriptase (Superscript™ III First-Strand Synthesis System; Life Technologies) was used to make cDNA from the castor RNA. Full-length transcripts were amplified from cDNA with KOD polymerase (Takara). The 5´ start primer contained the CACC sequence for directional topoisomerase cloning into pENTR-D-Topo (Life Technologies) see Supplementary Table 1 for the list of primers. The castor genes were then sequenced with vector primers (M13 forward and M13 reverse, Life Technologies) and compared with the castor genome at The Institute of Genomic Research (TIGR) castor genome database ( http://castorbean.tigr.org/) ^20^. These castor genes were renamed as follows: *RcCb5-1*, 28014.m000117; *RcCb5-2*, 30213.m000673; *RcCb5-3*, 29904.m002991; *RcCb5-4*, 30204.m001761; *RcCb5-5*, 29912.m005430; and *RcCb5-6*, 30174.m009087. From the entry vectors, these genes were subcloned into gateway compatible plant transformation vectors containing the seed specific β-phaseolin promoter, pGate-Phas-Basta (pGPB) or pGate-Phas-dsRed (pGPD), using LR Clonase I (Life Technologies). These constructs were then transformed into *Agrobacterium* GV3101 and used for plant transformations.

Dr. Edgar Cahoon (University of Nebraska-Lincoln) graciously provided a dual-gene plant transformation vector (renamed pEC-dsRed) along with a cassette vector pKMS2, which contains the seed-specific oleosin promoter. The pEC-dsRed vector contains two multiple cloning sites and the dsRed selection marker. This pEC-dsRed vector was made gateway compatible at the multiple cloning site containing the storage protein promoter glycinin. For cloning into pEC-dsRed, the *RcCBR1* gene was subcloned from pENTR into pCR-Script (Stratagene) with NotI restriction sites on both primers. *RcCBR1* gene out of pCR-Script and subclone it into the NotI site of pKMS2, which contains the oleosin promoter. The AscI sites were used to transfer the oleosin- *RcCBR1* cassette from pKMS2 and into pEC-dsRed. The pEC-dsRed-RcCBR1 was then used for three individual gateway reactions with each of the three RcCb5 entry vectors using LR Clonase I (Life Technologies), so that the oleosin promoter controlled the Rc *CBR1* expression and the glycinin promoter controlled the expression of the *RcCb5* genes. This cloning resulted in the three binary plant transformation vectors pEC-dsRed-RcCBR1-RcCb5-2, pEC-dsRed-RcCBR1-RcCb5-3, and pEC-dsRed-RcCBR1-RcCb5-4 that were transformed into the *Agrobacterium* strain GV3101.

### Transformations

Line CL37 ^13^ was transformed with constructs mentioned above using the floral dip method ^21^. In brief, a 72 h 500 mL culture of *Agrobacterium* containing the plasmid was resuspended in 5% sucrose and 0.05% Silwet L-77 (Lehle Seeds), flowers were dipped in the solution for 30 seconds, and plants were covered with plastic wrap over-night. For constructs containing the dsRed selection marker (pGPD and pEC-dsRed), T _1_ and T _2_ selection was conducted using a green LED light with a red filter ^13^. For the pGPB constructs, T _1_ transformants were selected for Basta® (Bayer) resistance on 1× MS-agar supplemented with 1% sucrose, containing 20 µg/mL glufosinate-ammonium.

### Fatty acid analysis

Fatty acid methyl esters were prepared from 20–50 seeds and analyzed by gas chromatography ^22^. Statistical analyses were conducted by an unpaired two-tailed *t*-test in Excel, with a 95% confidence (P < 0.05).

### Gene expression analysis

Ten to 12 developing siliques from select lines were frozen in liquid nitrogen and used for RNA extraction according to the protocol described by Onate-Sanchez and Vicente-Carbajosa ^23^, with minor modifications: tissue was ground in liquid nitrogen with a mortar and pestle and transferred into a pre-chilled microcentrifuge tube where 550 µL of extraction buffer (0.4 M LiCl, 0.2 M Tris pH 8, 25 mM EDTA, 1% SDS) and 550 µL of chloroform were added ^23^. The tubes were vortexed and then centrifuged for 3 min at 14,000 × g at 4°C. 500 µL of water-saturated acidic phenol, 200 µl of chloroform, and 8 µL of iso-amyl alcohol were added to the supernatant and the tubes were vortexed followed by centrifugation for 3 min. The supernatant was extracted twice with 500 µL of chloroform and 1/3 of a volume of 8 M LiCl was added to the resulting supernatant. The tubes were incubated at 20°C for 1.5 h and then centrifuged for 30 min at 14,000 × g at 4°C. The pellet was dissolved in 20 µL DEPC-water and subjected to DNaseI treatment according to the DNA-free RNA kit (Zymo). Complementary DNA was synthesized using Superscript III reverse transcriptase (Life Technologies) and the full-length castor genes were amplified using KOD polymerase (Takara), see Supplementary Table 1 for the list of primers.

## Results

### Identification of castor cytochrome b5 genes

In *Arabidopsis*, five cytochrome b5 proteins (AtCb5-A to AtCb5-E) and one AtCb5-like protein receive electrons from NADH:cytochrome b5 reductase encoded by *AtCBR1* ^24, 25^. In developing *Arabidopsis* seeds, the most highly expressed genes encode AtCb5-E (At5g53560) and AtCb5-D (At5g48810); *AtCb5-B* (At2g32720) is also strongly expressed, but there is only weak expression of the remaining three genes ^26, 27^. The *AtCb5-E* and *AtCb5-D* genes are also highly expressed in other tissues of the plant. Proteins encoded by these three strongly expressed isoforms are predicted to localize to the endoplasmic reticulum, based on their homology to Cb5 proteins from tung tree ( *Vernicia fordii*) VfCb5-A, VfCb5-B and VfCb5-C that have been shown to be targeted exclusively to the endoplasmic reticulum ^28^.

**Figure 1.**
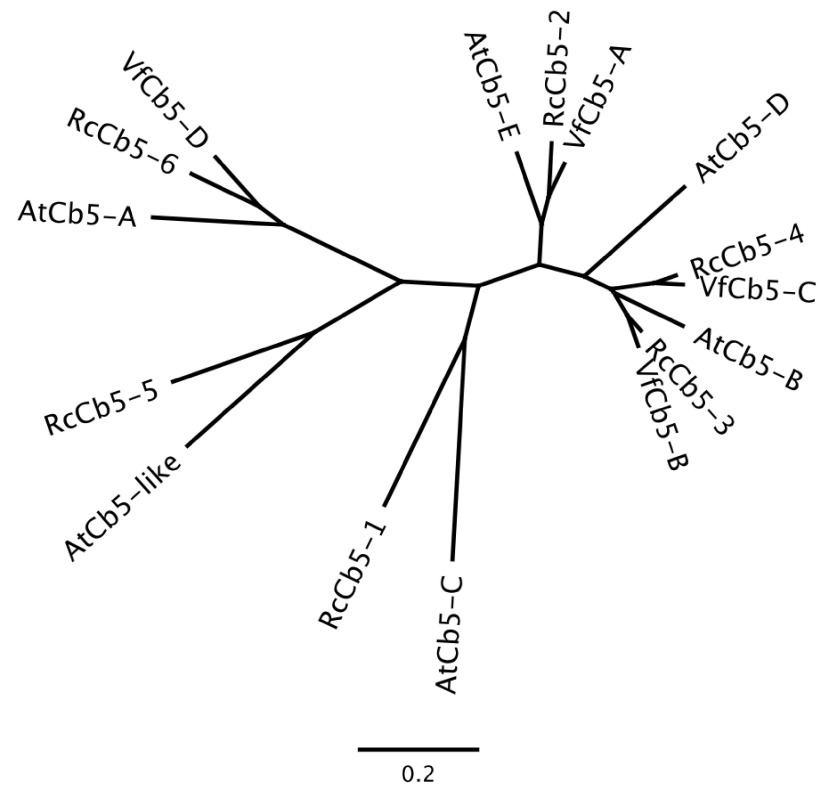
Phylogenetic relationships among 16 Cb5 proteins. Protein sequences from *Arabidopsis thaliana* (At), *Ricinus communis* (Rc), and *Vernicia fordii* (Vf) were used to build an unrooted dendrogram in Geneious v5.1 ( www.geneious.com). Size bar indicates genetic distance between proteins using the Neighbor-Joining method.

**Figure 2.**
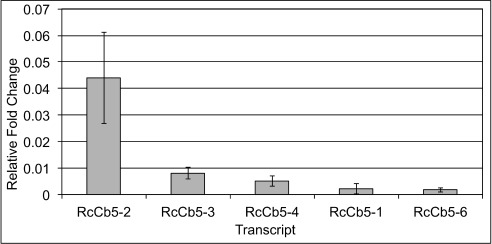
Quantitative PCR of *RcCb5* transcripts from developing castor endosperm. Transcripts were normalized to *RcACTIN*. Error bars represent standard error of the mean of three independent experiments with three replicates for each experiment.

The *Arabidopsis* and *Vernicia* protein sequences were used to search the castor genome ^20^ and identify likely RcCb5 orthologues. Sequences from all three species were used to derive an unrooted dendrogram ( Figure 1) showing the phylogenetic relationships among the proteins. The castor isoforms designated RcCb5-2, RcCb5-3 and RcCb5-4 (see Materials and methods for accession numbers) fall into a distinct clade with the *Vernicia* and *Arabidopsis* endoplasmic reticulum Cb5 proteins. Quantitative PCR (qPCR) was performed to measure the expression of *RcCb5* genes in developing endosperm of castor seeds during the period of HFA synthesis in this tissue ^19^. We found that *RcCb5-2*, *RcCb5-3* and *RcCb5-4* transcripts were all strongly expressed, with highest transcript levels found for *RcCb5-2* ( Figure 2). By contrast, the *RcCb5-1* and *RcCb5-6* genes showed very low expression. Data from a transcript profiling database with ~10 ^6^ sequences covering five stages of endosperm development ^29^ includes 21 reads for *RcCb5-2* and 26 reads for *RcCb5-4*, while *RcCb5-3*, *RcCb5-1* and *RcCb5-6* were not represented in this data set. Taken together, these results indicate that RcCb5-2, RcCb5-3 and RcCb5-4 likely have redundant roles in providing electrons to the FAH12 hydroxylase enzyme in the endoplasmic reticulum of endosperm cells of developing castor seeds.

### Transformation of the CL37 line with three RcCb5 proteins

**Figure 3.**
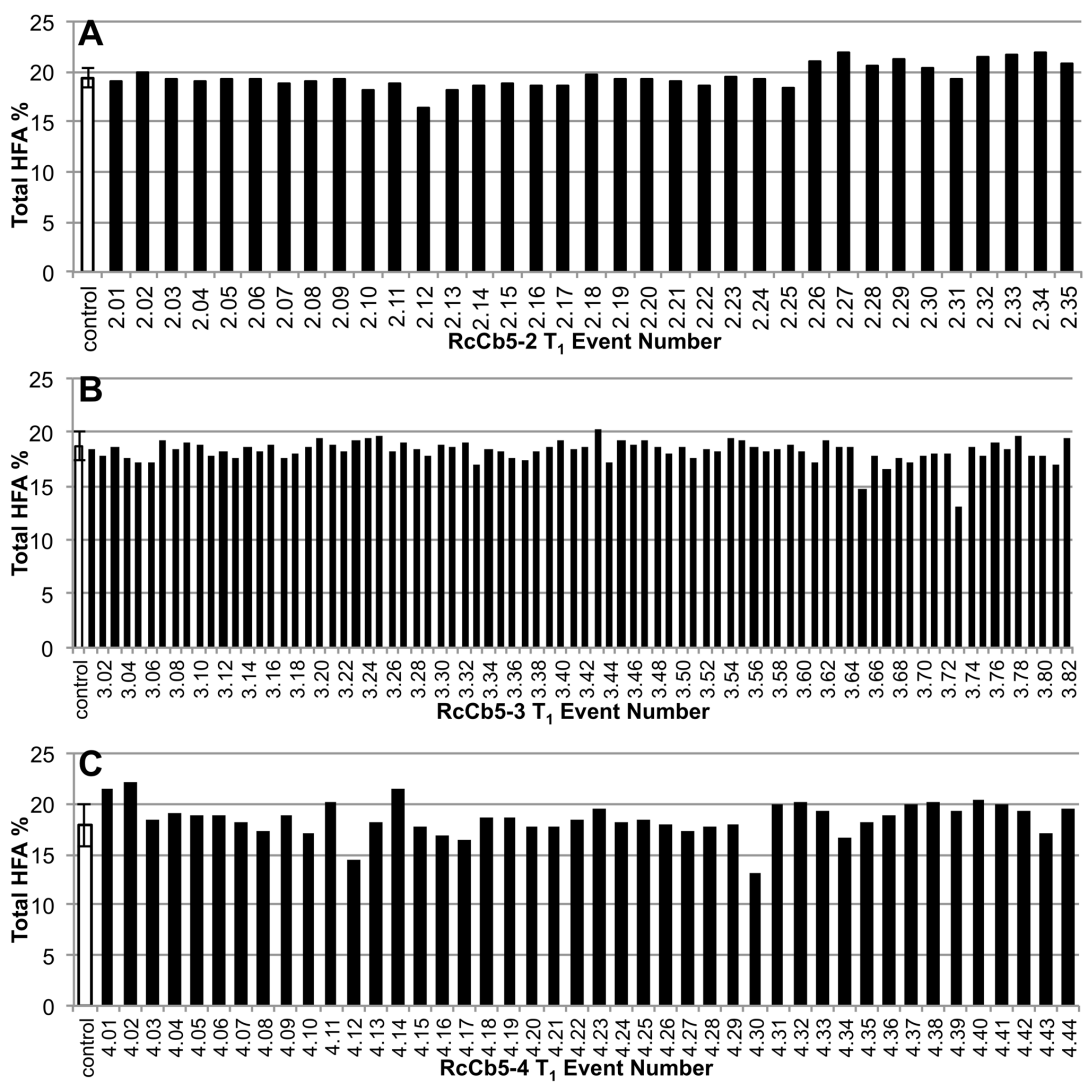
Total HFA accumulation in seed of individual RcCb5 CL37 primary transformants. Total HFA (18:1-OH+18:2-OH) contents of mature T _2_ seeds of individual transformants expressing ( **A**) RcCb5-2, ( **B**) RcCb5-3, or ( **C**) RcCb5-4 in the CL37 line. Control at left is mean HFA in untransformed CL37 plants grown alongside T _1_ plants. For controls, error bars indicate standard deviation; n=28 in **A**, n=11 in **B**, and n=15 in **C**.

The cDNAs encoding the three RcCb5 isoforms that are strongly expressed in seeds (RcCb5-2, RcCb5-3 and RcCb5-4) were each cloned under control of the phaseolin promoter in vector pGate-Phas-Basta and transformed into CL37 plants expressing RcFAH12 ^13^. Putative T _1_ transformants were selected for Basta resistance. Analysis of the bulk T _2_ seed from 35 independent RcCb5-2 CL37 primary transformants showed no significant difference in total HFA (18:1-OH plus 18:2-OH) levels compared to the control CL37 plants grown at the same time, with a mean of 19.5% HFA (P value = 0.5979) for the 35 RcCb5-2 events ( Figure 3A). There were also no significant differences in HFA levels for the 82 independent T _1_ events for RcCb5-3 CL37 compared to untransformed CL37, with a mean of 18.3% HFA (P value = 0.2155) ( Figure 3B), nor for the 44 independent T _1_ events of RcCb5-4 CL37 (mean of 18.6% HFA) compared to untransformed CL37 (P value = 0.2470; Figure 3C).

**Figure 4.**
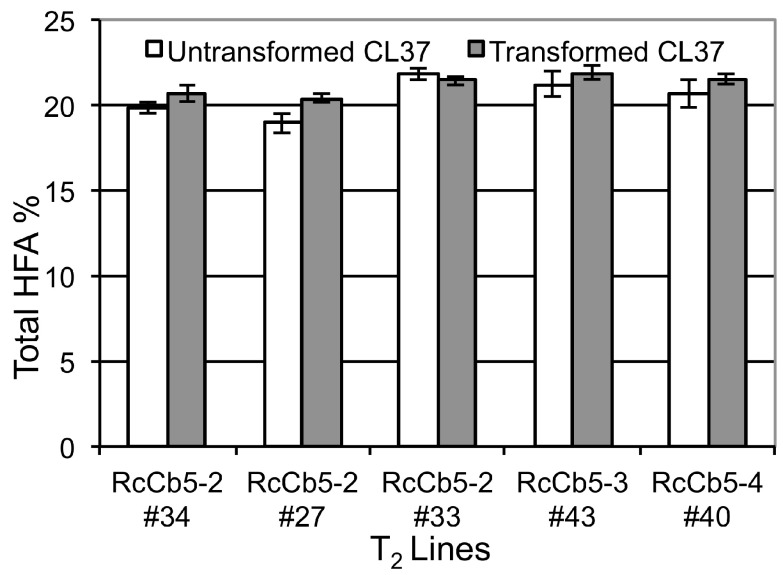
Total HFA accumulation in RcCb5 CL37 T _3_ seed from selected T _2_ lines. Total HFA (18:1-OH+18:2-OH) contents of T _3_ seed of selected homozygous lines expressing RcCb5-2, RcCb5-3, or RcCb5-4 in CL37 were compared with segregating untransformed CL37 siblings. Error bars represent standard error of the mean for four to ten individual plants.

This analysis of T _2_ seed samples indicates that there is no dramatic increase in HFA accumulation through the co-expression of any of the three RcCb5 isoforms with the RcFAH12 in *Arabidopsis*. However, gene expression is typically variable across a population of individual T _1_ transformants and T _2_ seeds are segregating for the newly introduced transgene. We therefore identified five T _1_ events that produced seed with relatively high HFA and that contained a single *RcCb5* transgene insert, based on segregation of the Basta-resistance marker in the T _2_ generation. For each of the chosen lines, RcCb5-2 #34, RcCb5-2 #27, RcCb5-2 #33, RcCb5-3 #43, and RcCb5-4 #40, T _2_ progeny were grown to maturity and nine to 10 plants homozygous for the *RcCb5* transgenes, together with four to nine segregants lacking the transgene from each event, were identified by pedigree analysis of T _3_ progeny for Basta resistance. Comparisons of the total HFA content between these two sets of sibling segregants ( Figure 4) failed to identify any statistically significant increase in HFA as a result of *RcCb5* expression. Thus, plants from the three lines of RcCb5-2 CL37, #34, #27, and #33, were not significantly different from their segregating untransformed CL37 counterparts, with P = 0.2204, 0.0942, and 0.3965 respectively ( Figure 4). The RcCb5-3 CL37 #43 was not significantly different from segregating untransformed CL37 (P = 0.4211), and the RcCb5-4 CL37 #40 was also not significantly different from segregating untransformed CL37 (P = 0.2102) ( Figure 4). Although no changes were observed in the total HFA levels from these lines, we did observe some minor changes in the fatty acid profile; there were increases in the level of 18:1 and minor decreases in 18:2 and 18:3 ( Supplementary Table 2).

**Figure S1.**
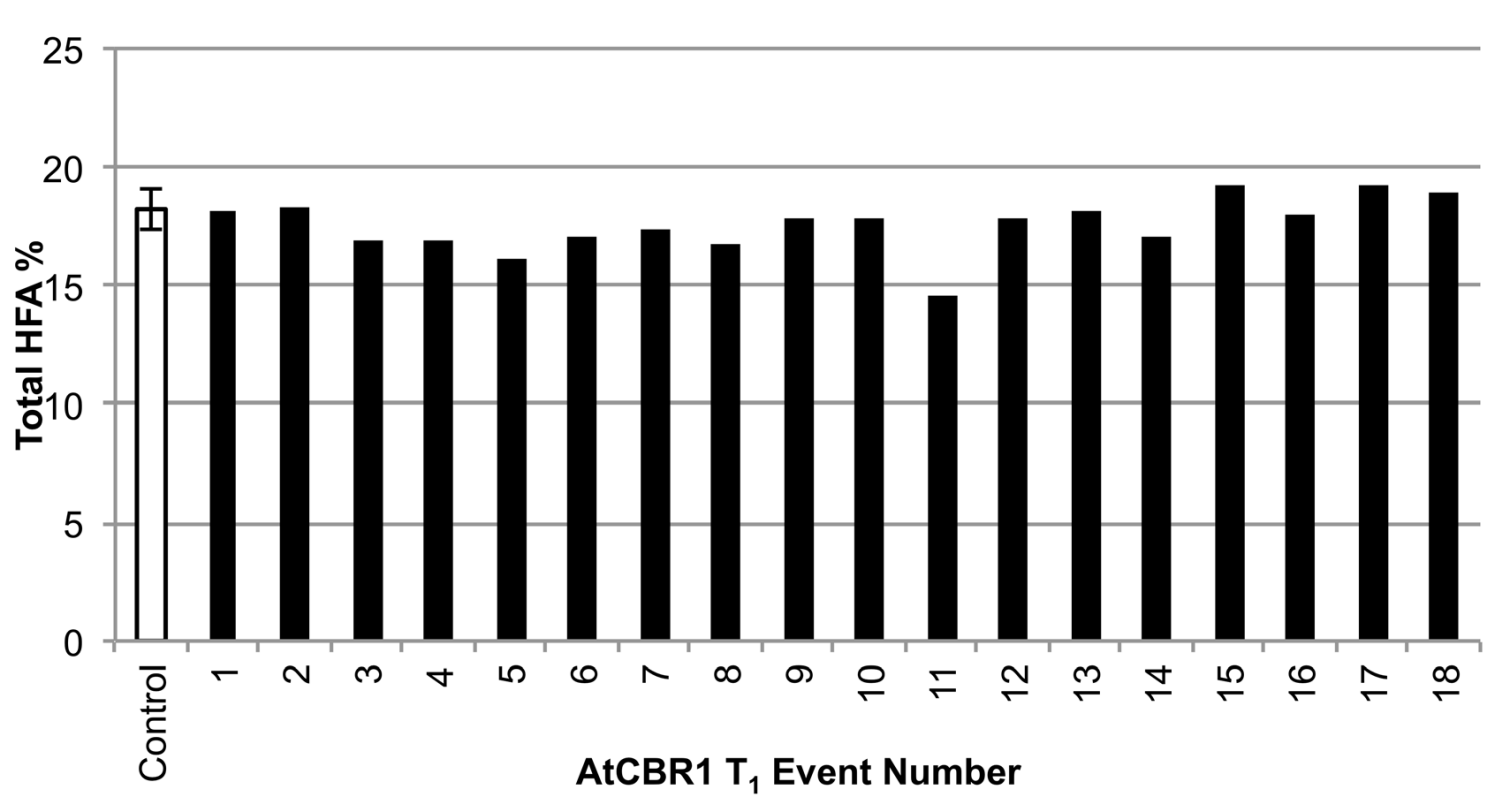
Total HFA accumulation in AtCBR1 CL37 primary transformants. Total HFA (18:1-OH+18:2-OH) contents of mature T _2_ seeds of individual transformants expressing AtCBR1 in CL37. Control at left is mean HFA in untransformed CL37 plants grown alongside the T _1_ plants. For the control, error bar indicates standard deviation, n=12.

Since it is assumed that the RcFAH12 in the CL37 line is receiving reductant via the endogenous *Arabidopsis* CBR1 (AtCBR1) and an AtCb5, we attempted to increase reductant supply to the AtCb5 proteins via overexpressing *AtCBR1*. However, again we found no changes in total HFA in the T _2_ seeds ( Supplementary Figure 1). Taken together, these results indicate that none of the three RcCb5 proteins or any of the AtCBR1 electron acceptors can provide increased RcFAH12 activity in CL37.

### Coexpression of RcCBR1 and RcCb5s in CL37

**Figure 5.**
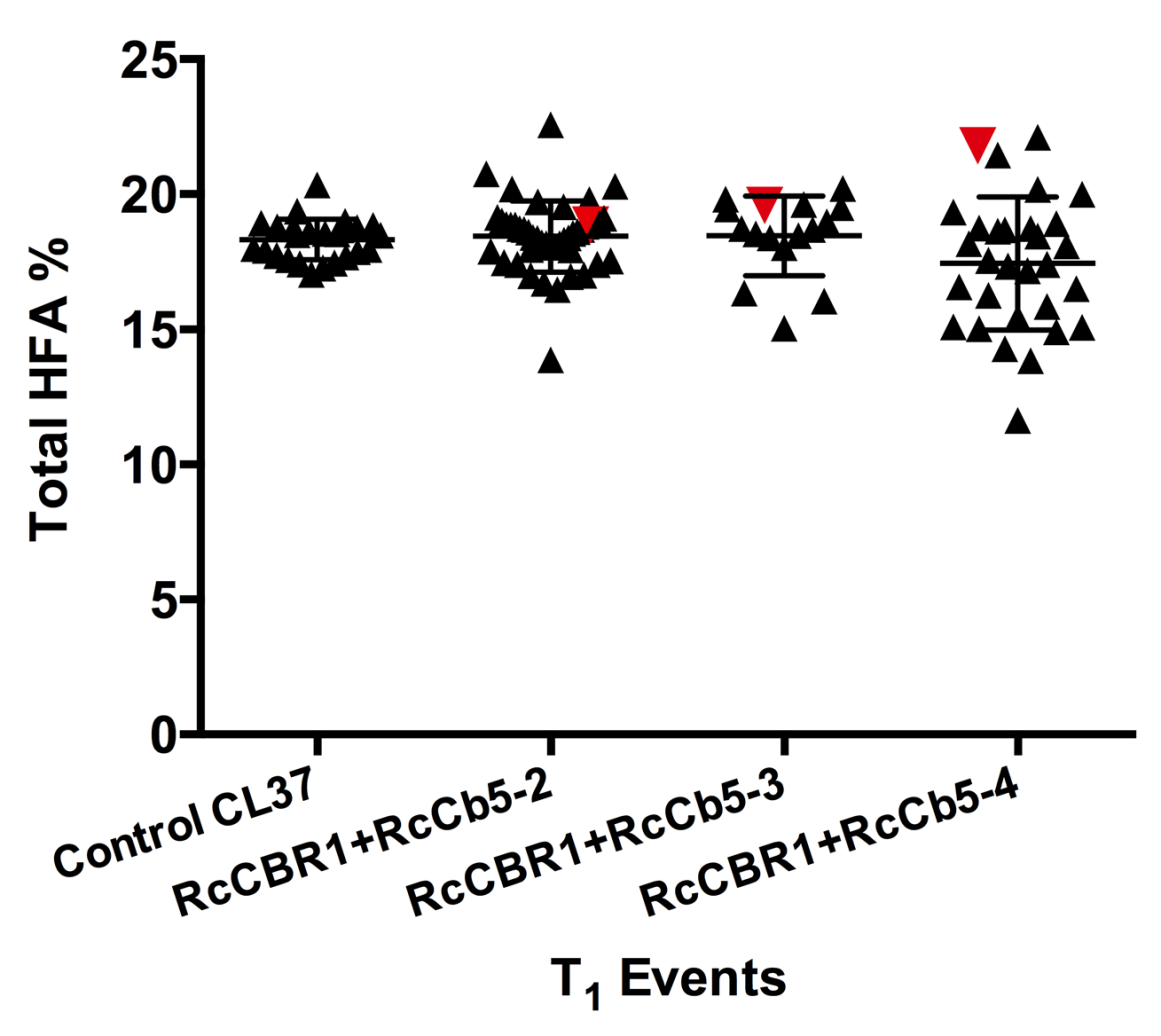
Total HFA accumulation in seed of individual RcCBR1+RcCb5 CL37 primary transformants. Total HFA (18:1-OH+18:2-OH) contents of mature T _2_ seeds from individual transformants expressing RcCBR1+RcCb5-2, RcCBR1+RcCb5-3 or RcCBR1+RcCb5-4 in the CL37 line. Results are compared with untransformed CL37 controls grown alongside T _1_ plants. Red inverse triangles indicate events that were further analyzed. Error bars represent standard deviation; n=25 for control CL37, n=45 for RcCBR1+RcCb5-2, n=16 for RcCBR1+RcCb5-3, and n=30 for RcCBR1+RcCb5-4.

We transformed CL37 plants with *RcCb5* genes on the basis that the RcFAH12 hydroxylase enzyme may not interact optimally with endogenous AtCb5 proteins to receive electrons required for the hydroxylation reaction. However, it is also possible that interactions of the RcCb5 isoforms with the *Arabidopsis* cytochrome b5 reductase (AtCBR1) are weak, resulting in incomplete reduction of the RcCb5 proteins in the transgenic plants. To test this possibility, and to provide a fully compatible electron transport chain from NADH to the hydroxylase, we transformed CL37 plants with expression constructs containing both *RcCBR1*, encoding the castor cytochrome b5 reductase isozyme and a *RcCb5* coding sequence, under control of strong, seed-specific promoters (see Materials and methods for details). The *RcCBR1* and each of the top three *RcCb5* genes were subcloned into pEC-dsRed plant transformation vector, resulting in three dual-gene vectors expressing *RcCBR1*+ *RcCb5-2*, *RcCBR1*+ *RcCb5-3*, and *RcCBR1*+ *RcCb5-4*. The T _1_ transformants were selected by screening for dsRed fluorescence in seeds and grown to maturity. Screening of individual transformation events for total HFA accumulation was performed by gas-chromatography of bulk T _2_ seeds. The 45 RcCBR1+RcCb5-2 CL37 events generated were not statistically different (mean of 18.4% HFA) from the untransformed CL37 control lines (P = 0.6820) ( Figure 5). For the 16 RcCBR1+RcCb5-3 CL37 events the mean of 18.5% HFA was not significantly changed in HFA accumulation compared to the CL37 control plants (P = 0.6834). Similarly, the 30 RcCBR1+RcCb5-4 CL37 events (mean of 17.4% HFA) also were not significantly different from the CL37 controls (P = 0.0898).

**Figure 6.**
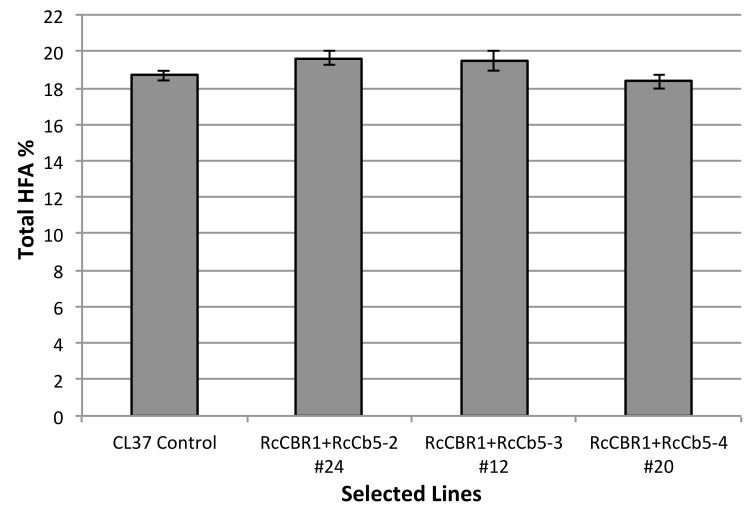
Total HFA accumulation in RcCBR1+RcCb5 CL37 from selected lines. Total HFA (18:1-OH+18:2-OH) contents of seed from selected lines expressing RcCBR1+RcCb5-2, RcCBR1+RcCb5-3 or RcCBR1+RcCb5-4 in CL37. Plants homozygous for each dual-gene construct were compared with untransformed CL37 controls. Error bars represent standard error of the mean; n=14 for CL37 controls, n=6 for #24, n=7 for #12, and n=4 for #20.

Three single-insert lines with relatively high HFA were selected from the T _1_ events shown in Figure 5. Progeny from each of the three lines were grown to maturity and four to seven plants homozygous for the *RcCBR1* and *RcCb5* transgenes were identified by the presence of the DsRed marker in their seeds and were grown together with CL37 control plants. Analyses of total HFA content of the homozygous seed showed no statistically significant difference between RcCBR1+RcCb5-2 #24 double transgenic and seed of CL37 lacking these transgenes (P = 0.0571; Figure 6). Similarly, HFA in seed of homozygous plants of lines RcCBR1+RcCb5-3 #12 and RcCBR1+RcCb5-4 #20 were not statistically different from HFA in CL37 controls (P = 0.1388 and P = 0.5774 respectively; Figure 6).

**Figure 7.**
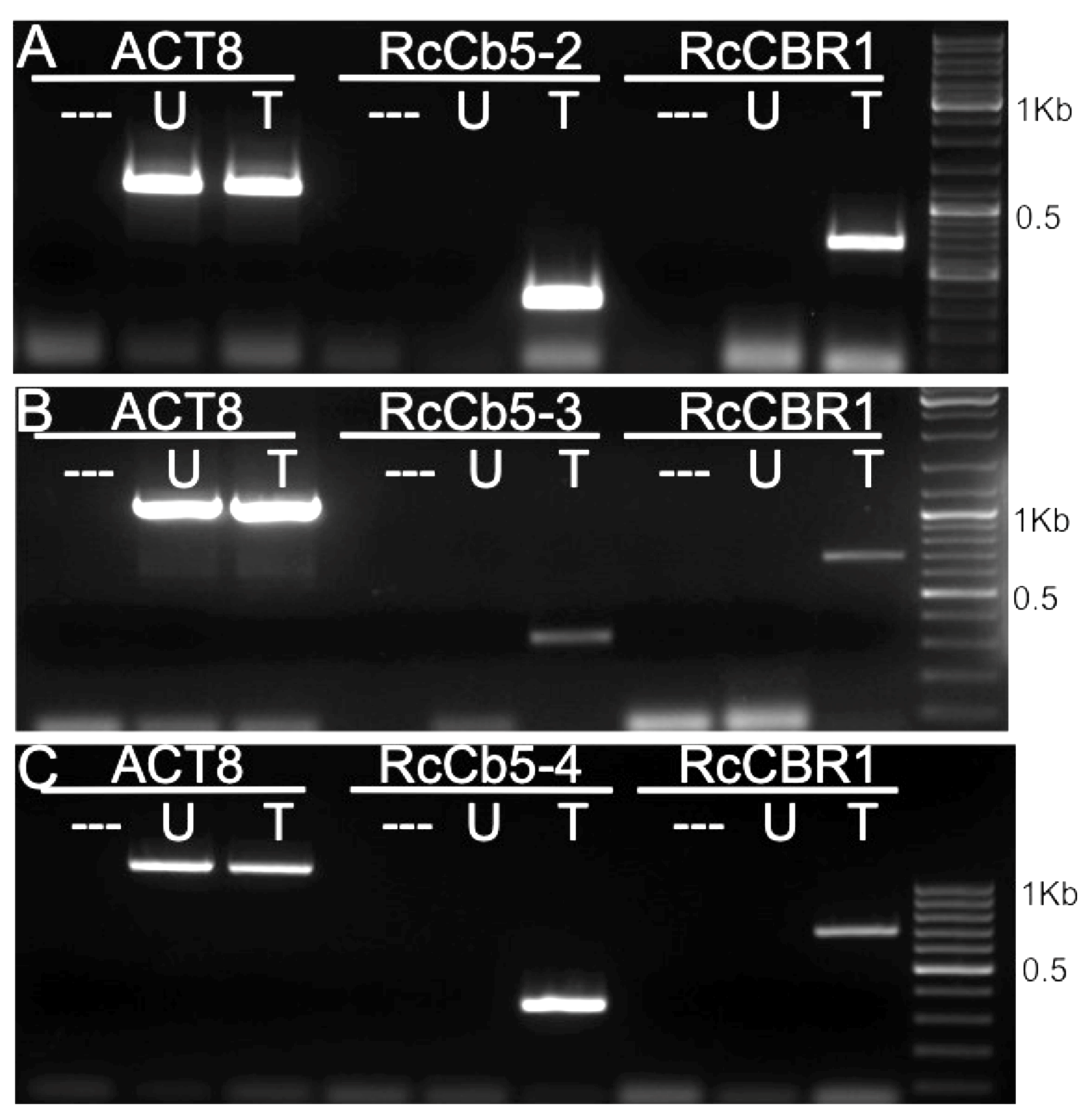
Reverse transcriptase-PCR from representative RcCBR1+RcCb5 CL37 T _3_ plants. ( **A**) Rc *CBR1*+Rc *Cb5-2* #24, ( **B**) Rc *CBR1*+Rc *Cb5-3 #12* and ( **C**) Rc *CBR1*+Rc *Cb5-4* #20. RNA prepared from developing siliques was subject to RT-PCR using gene-specific primers. In each panel, --- indicates no-template control. U indicates untransformed CL37, T indicates homozygous transformant. The *ACTIN8* gene ( *ACT8*, At1g49240) was used as a control to test RNA quality.

To confirm that the *RcCBR1* and *RcCb5* transgenes were being expressed, we isolated RNA from developing seeds of representative plants from these three homozygous transgenic lines, and from the untransformed CL37 siblings. The RNA samples were used as templates for RT-PCR using primers specific for *RcCBR1* and each of the *RcCb5* isoforms. Bands of the expected full-length sizes were observed for the transgenic plants but were not detected in the untransformed CL37 siblings ( Figure 7). Successful amplification of a band using primers to the *ACT8* gene indicated that none of the RNA samples was degraded.

## Discussion

The endoplasmic reticulum diiron enzymes that catalyze desaturation and hydroxylation of fatty acids and other substrates require electrons from cytochrome b5(Cb5) proteins ^6, 30^. In seeds of transgenic *Arabidopsis* expressing the RcFAH12 hydroxylase from castor, a hypomorphic mutation in the *CBR1* gene encoding cytochrome b5 reductase led to an 85% reduction in total HFA, but had smaller effects on 18:1 and 18:2 desaturation, <25% and <60% respectively ^15^. These results indicated that there is competition among the reactions that utilize electrons from reduced Cb5, and raised the possibility that the RcFAH12 enzyme is not able to efficiently accept electrons from the endogenous AtCb5 proteins. In another study, we have shown that the activity of a *T. thermophila* desaturase expressed in yeast is strongly limited by the failure of the desaturase to properly interact with, and receive electrons from, the yeast Cb5. A nearly ten-fold increase in desaturase activity was achieved by coexpressing *T. thermophila* Cb5 proteins ^18^.

When RcFAH12 is heterologously expressed in *Arabidopsis*, the reductant supply for this enzyme comes from the endogenous *Arabidopsis* proteins. Here, we have tested the proposal that, since the castor orthologs of CBR1 and Cb5 have coevolved with RcFAH12, these proteins may be more efficient at supplying reductant to RcFAH12 and lead to a greater accumulation of HFA. The coevolution hypothesis has been shown to be valid for two castor acyltransferases, RcDGAT2 and RcPDAT1A; when these castor enzymes were heterologously expressed with the RcFAH12 in *Arabidopsis*, the HFA content of seeds increased from 17% to as much as 30% of total fatty acids, due to the increased efficiency of HFA incorporation into TAG ^22, 31^. However, contrary to our hypothesis, we found that there was no substantial increase in HFA accumulation when any of three RcCb5 proteins were expressed in CL37 ( Figure 3 and Figure 4). These results argue against there being a substantial difference in the effectiveness of interaction of RcFAH12 with RcCb5 versus AtCb5 protein isoforms. Overexpressing the AtCBR1 in CL37 also did not increase HFA levels ( Supplementary Figure 1), indicating that reductant supply via AtCBR1 is not limiting to the hydroxylase activity. However, these experiments cannot exclude the possibility that AtCBR1 does not efficiently reduce RcCb5 proteins.

To reconstitute the complete electron transport chain from NADH to the hydroxylase present in castor seeds, we transformed *RcCBR1* and *RcCb5* genes into the CL37 line, using a dual-gene plant expression system. The proportion of HFA in seeds of plants expressing RcCBR1+RcCb5-2 CL37, RcCBR1+RcCb5-3 CL37, or RcCBR1+RcCb5-4 CL37 was not significantly increased in comparison to the parental CL37 line. Although we confirmed expression of *RcCBR1* and *RcCb5* transcripts ( Figure 7), production of functional reductase and cytochrome b5 requires translation, correct protein folding in the endoplasmic reticulum, and insertion of the required cofactor. It is possible that either RcCBR1 or (all of) the Cb5 proteins fail to assemble to the mature form. Assuming the proteins are correctly expressed, our results suggest that electron transfer to RcFAH12 is not limiting in the CL37 line, and that the endogenous *Arabidopsis* electron transfer system appears to be as efficient in transferring electrons to RcFAH12 as the transgenic castor electron transfer system in *Arabidopsis*.

Taken as a whole, our results fail to show any positive effect of expressing genes of the castor electron-transport components on the synthesis and accumulation of HFA in transgenic *Arabidopsis* expressing the castor RcFAH12 hydroxylase. However, our CL37 transgenics only accumulate 17–19% HFA in the seed oil, compared to almost 90% HFA found in oil from castor ^22, 31^. Some of the constraints on HFA synthesis and accumulation have been identified ^22, 32, 33^. If these constraints can be alleviated, by more efficient shuttling of HFA into triacylglycerol or reducing feedback inhibition of metabolism for example, it is possible that electron supply to the hydroxylase will then become limiting. If so, the constructs and lines generated in this study can be used for transforming and crossing with the elite lines containing higher levels of HFA.

Fatty acid hydroxylation in transgenic Arabidopsis thaliana RcCb5-2 T1 events.csv Hydroxy fatty acid (HFA) percent from T2 seed from T1 events of RcCb5-2 transformed into CL37. Data are presented in Figure 3A.
RcCb5-3 T1 events.csv Hydroxy fatty acid (HFA) percent from T2 seed from T1 events of RcCb5-3 transformed into CL37. Data are presented in Figure 3B.
RcCb5-4 T1 events.csv Hydroxy fatty acid (HFA) percent from T2 seed from T1 events of RcCb5-4 transformed into CL37. Data are presented in Figure 3C.
RcCBR1 RcCb5s T1 events.csv Hydroxy fatty acid (HFA) percent from T2 seed from T1 events of RcCBR1+RcCb5 transformed into CL37. Data are presented in Figure 5.
AtCBR1 T1 events.csv Hydroxy fatty acid (HFA) percent from T2 seed from T1 events of AtCBR1 transformed into CL37. Data are presented in Supplementary Figure 1.
RcCb5s T2 lines.csv Hydroxy fatty acid (HFA) percent from T3 seed from T2 events of RcCb5s transformed into CL37. Data are presented in Figure 4 and Supplementary Table 1. Please note that a colon “:” is normally used in the x:y (number of carbon:number of double bonds) nomenclature of the fatty acids, however to avoid the use of the colon in this dataset we have the carbon number followed by the letter “c” and then number of double bonds. For example “18c 1” (18:1) is oleic acid.
RcCBR1 RcCb5 selected lines.csv Hydroxy fatty acid (HFA) percent from T3 seed from T2 events of RcCBR1+RcCb5s transformed into CL37. Data are presented in Figure 6.
Q-PCR data.csv Quantitative reverse-transcriptase-PCR of developing castor bean endosperm. Transcripts were normalized to RcACTIN. Error bars represent standard error of the mean of three independent experiments with three replicates each. Relative fold change = 1/(2^(experimental-control). Data are presented in Figure 2.

## Figures and Tables

**Table S1.** List of primers used in experiments.

Experiment	Primers	Sequence
Q-PCR	RcCb5-2 QFW RcCb5-2 QRV RcCb5-4 QFW RcCb5-4 QRV RcCb5-3 QFW RcCb5-3 QRV RcCb5-1 QFW RcCb5-1 QRV RcCb5-6 QFW RcCb5-6 QRV RcACTIN QFW RcACTIN QRV	AAACTTCGAAGAGGTGGCTAA TGTAGTTGGCTTGTGGTTTAGG GGGTGGTCAAGGAAAGGTTTAT GAGGCTGCTTAGGAGGAGTATATG GTTTTCACTTTGGCTCAGGTGT GGCTTATATGTCGCCTTCTTAGG GGCTTCAGAACCCAAAATCT GGTTCTTAGGATGTTCTGAAGGTG GCTCAGCACAACACCAAAGAG CTTCGTCAAGTCCTTGAGCTTC AAGGTTATGCTCTCCCACATGCCA ACTTCTGGGCAACGGAATCTCTCA
Cloning & RT-PCR	RcCb5-2 5topo RcCb5-2 3end RcCb5-4 5topo RcCb5-4 3end RcCb5-3 5topo RcCb5-3 3end	CACCATGGCGAGCGATCCGAAAAT CTACTCTTTCTTGGTGTAGTGCCTGAC CACCATGGGTGGTCAAGGAAAGGTTTATA CTATTATGCTTCAGCTGATTTGGTGT CACCATGAGTGGTGAAGGCAAAGTTT TTAAGTTGATTTTGTATAGATGCGGAT
Sub-cloning	RcCBR1 5´Not1 RcCBR1 3´Not1	CACCGAATGCGGCCGCATGGATTTAGATT TCTTGCAAACAC ATAGTTTAGCGGCCGCTCAAAATTGGAACT GCATCTCA

**Table S2.** Fatty acid profile from RcCb5 CL37 T _3_ seed from selected T _2_ lines.

	16:0	16:1	18:0	18:1	18:2	18:3	20:1	18:1-OH	18:2-OH	Total HFA ^a^
RcCb5-2 #34	10.8%	0.5%	4.3%	44.0%	13.8%	5.3%	0.6%	14.9%	5.8%	20.7%
n=10	0.3%	0.0%	0.2%	0.6%	0.4%	0.1%	0.0%	0.2%	0.4%	0.5%
Untransformed #34	12.5%	0.6%	5.2%	34.7%	19.5%	6.8%	0.8%	15.4%	4.5%	19.9%
n=9	0.4%	0.0%	0.2%	0.8%	0.5%	0.1%	0.0%	0.2%	0.3%	0.3%
RcCb5-2 #27	11.1%	0.5%	4.8%	44.6%	13.2%	4.5%	0.8%	15.5%	4.9%	20.4%
n=9	0.3%	0.0%	0.2%	0.9%	0.5%	0.6%	0.0%	0.4%	0.2%	0.3%
Untransformed #27	13.3%	0.6%	5.5%	33.2%	20.9%	6.7%	0.9%	14.8%	4.1%	18.9%
n=6	0.7%	0.0%	0.3%	1.3%	0.7%	0.1%	0.1%	0.3%	0.4%	0.5%
RcCb5-2 #33	10.3%	0.6%	4.8%	45.3%	12.0%	4.8%	0.7%	16.2%	5.3%	21.4%
n=10	0.3%	0.0%	0.2%	1.2%	0.8%	0.2%	0.0%	0.3%	0.5%	0.2%
Untransformed #33	11.0%	0.5%	4.8%	39.9%	15.7%	5.7%	0.7%	16.5%	5.3%	21.8%
n=8	0.1%	0.1%	0.2%	1.3%	1.4%	0.3%	0.0%	0.4%	0.3%	0.4%
RcCb5-3 #43	12.4%	0.6%	5.5%	38.5%	15.3%	5.0%	0.8%	17.6%	4.3%	21.9%
n=10	0.3%	0.0%	0.2%	1.1%	0.6%	0.6%	0.0%	0.2%	0.2%	0.4%
Untransformed #43	13.0%	0.6%	5.5%	36.9%	17.5%	4.5%	0.8%	16.5%	4.8%	21.2%
n=9	1.1%	0.0%	0.5%	1.7%	1.2%	0.8%	0.1%	0.3%	0.5%	0.8%
RcCb5-4 #40	11.6%	0.6%	4.7%	39.4%	16.6%	5.0%	0.6%	16.4%	5.1%	21.5%
n=10	0.3%	0.0%	0.2%	0.4%	0.4%	0.5%	0.0%	0.2%	0.3%	0.3%
Untransformed #40	13.7%	0.7%	6.1%	35.2%	18.2%	4.6%	0.9%	16.8%	3.8%	20.7%
n=4	0.6%	0.0%	0.3%	1.2%	0.9%	1.5%	0.1%	0.6%	0.3%	0.8%

^a^Total HFA is the sum of 18:1-OH and 18:2-OH.

Standard error of the mean is below each value.
