# Supplementary material for: Homologous electron transport components fail to increase fatty acid hydroxylation in transgenic Arabidopsis thaliana
Source: F1000Res. 2013 Nov 13;2:203. Originally published 2013 Oct 4. [Version 2] doi: 10.12688/f1000research.2-203.v2 (PMC3893003; doi:10.12688/f1000research.2-203.v2)
Supplement: Peer review history

# Peer review: Referee response for version 2

**DOI:** 10.5256/f1000research.2955.r3175

**Published:** 2014-01-17

**Authors:** Mark Smith

**Affiliations:** 1 National Research Council Canada, Saskatoon, SK, Canada

I agree with the authors response to my comments, the evidence supports the conclusion that expression of genes encoding homologous electron transport components does not appear to be of value in this case. Further work would provide an improved characterization of the transgenic plants, but the conclusion would most likely remain the same. This is an interesting study and adds some valuable information to plant lipid research.

I have read this submission. I believe that I have an appropriate level of expertise to confirm that it is of an acceptable scientific standard.

# Peer review: Referee response for version 2

**DOI:** 10.5256/f1000research.2955.r2440

**Published:** 2013-11-18

**Authors:** Daniel Gallie

**Affiliations:** 1 Department of Biochemistry, College of Natural and Agricultural Sciences, University of California, Riverside, CA, USA

I have no comments other than those I made for the first version.

I have read this submission. I believe that I have an appropriate level of expertise to confirm that it is of an acceptable scientific standard.

# Peer review: Referee response for version 1

**DOI:** 10.5256/f1000research.2397.r2243

**Published:** 2013-11-06

**Authors:** Mark Smith

**Affiliations:** 1 National Research Council Canada, Saskatoon, SK, Canada

FAD2 family proteins in plants require a reductant that is channelled via an electron transport chain comprising of cytochrome b5 (Cb5) and cytochrome b5 reductase (CBR). This study explores the hypothesis that lower than expected levels of hydroxy fatty acid (HFA) accumulation in engineered Arabidopsis could in part be due to inefficient interaction between the expressed castor hydroxylase and the endogenous Cb5 electron transport chain. The authors used a well characterized line of Arabidopsis (CL37) previously engineered to express the castor hydroxylase. Retransformation with a various cDNAs encoding Cb5 or Cb5+CBR1 failed to generate lines with increased HFA content.

The study addresses an important and somewhat overlooked aspect of the ER fatty acid modification processes. The experiments are well planned, and the comparison of null and transgenic lines in single insertion lines (Fig.4) adds confidence to the data. My only concern is that although there is evidence of transgene expression by RT-PCR, there is no evidence to show the assembly of a functional electron transport chain containing the expressed proteins, or an increase in electron transport activity resulting from Cb5 and CBR overexpression. A biologically active Cb5 requires the assembly of cytochrome b5 apoprotein and haem to generate the Cb5-holoprotein. Demonstrating over-expression of active Cb5 would require a spectrophotometric assay of microsomal membranes to quantify the amount of Cb5 present and to determine electron transport activity (not easy in Arabidopsis). Alternatively, a Western blot showing increased Cb5 protein and a good argument that haem-protein assembly in developing Arabidopsis seeds is not limiting would help.

An added complication from measuring total HFA in mature seed is that changes in HFA production could be masked by bottlenecks in HFA assembly into TAG. 

Further development of this work will be very interesting; the conclusions would be better supported by characterization of the electron transport chain.

I have read this submission. I believe that I have an appropriate level of expertise to confirm that it is of an acceptable scientific standard.

# Response

**Published:** 2013-11-07

**Authors:** John Browse

**Affiliations:** Institute of Biological Chemistry, Washington State University, USA

Mark Smith is correct in pointing out that in biochemistry (and it life) it is protein function that matters. We have added a caveat on p.10 to acknowledge the possibilities he raises.

Unfortunately, microsomal membranes from Arabidopsis seeds are contaminated with chlorophyll-containing thylakoids and the pigment confounds the spectrophotometric measurement of cytochrome b5. Even with the possibility that redox-competent RcCb5 and RcCBR1 are not produced, our conclusion remains the same: Expression of genes encoding homologous electron-transport components does not present itself as a useful route to increasing HFA accumulation in transgenic plants.

# Peer review: Referee response for version 1

**DOI:** 10.5256/f1000research.2397.r1998

**Published:** 2013-10-07

**Authors:** Daniel Gallie

**Affiliations:** 1 Department of Biochemistry, College of Natural and Agricultural Sciences, University of California, Riverside, CA, USA

The castor fatty acid hydroxylase (RcFAH12) is responsible for the synthesis of ricinoleic acid which has important industrial applications. Heterologous expression of RcFAH12 in *Arabidopsis thaliana* resulted in only 17-19% hydroxylated fatty acid (HFA) in seed triacylglycerols (TAG). In this work, the authors suggest that electron supply to RcFAH12 in *Arabidopsis* is limiting due to its heterologous expression and they test this hypothesis by co-expressing castor NADH:cytochrome b5 reductase (RcCBR1) and cytochrome b5 (RcCb5) that supply electrons to RcFAH12.

The work is well presented and performed and indicates that the endogenous electron transfer system in Arabidopsis is not rate limiting in HFA production.

I have read this submission. I believe that I have an appropriate level of expertise to confirm that it is of an acceptable scientific standard.
